# Simultaneous Native Nephrectomy and Kidney Transplantation in Patients With Autosomal Dominant Polycystic Kidney Disease

**DOI:** 10.1371/journal.pone.0155481

**Published:** 2016-06-03

**Authors:** Massimiliano Veroux, Domenico Zerbo, Giusi Basile, Cecilia Gozzo, Nunziata Sinagra, Alessia Giaquinta, Angelo Sanfiorenzo, Pierfrancesco Veroux

**Affiliations:** Vascular Surgery and Organ Transplant Unit, Department of Medical and Surgical Sciences and Advanced Technologies GF Ingrassia, University Hospital of Catania, Via Santa Sofia, 84, 95123, Catania, Italy; University of Catania, ITALY

## Abstract

**Introduction:**

To evaluate the feasibility of simultaneous unilateral nephrectomy with kidney transplantation and to determine the effect of this procedure on perioperative morbidity and mortality and graft and patient survival.

**Methods:**

Between January 2000 and May 2015, 145 patients with autosomal dominant polycystic kidney disease (ADPKD) underwent kidney transplantation. Of those, 40 (27.5%) underwent concurrent ipsilateral native nephrectomy (group NT). Patients in group NT were compared with patients with ADPKD not undergoing concurrent nephrectomy (group NT-) and asymptomatic patients undergoing pretransplant nephrectomy (group PNT).

**Results:**

The average follow-up was 66 months. The graft survival rate at 1 and 5 years was 95% and 87.5% versus 93% and 76.2% in the NT and NT- groups, respectively (*P =* .903 and *P* = .544, respectively); 1-year patient survival was 100% for NT and 97% for NT- patients (*P =* .288), whereas 5-year patient survival was 100% and 92% for NT and NT- groups, respectively (*P =* .128). After propensity score matching (34 patients per group) no significant differences were observed in 1-year (97.1% in NT and 94.1%; *P* = 1) and 5-year (88.2% in NT and 91.2% in NT-; *P* = 1) graft survival, and in 1-year (100% for both groups; *P* = 1) and 5-year (100% in NT and 94.1% in NT-; *P* = 1) patient survival. Perioperative mortality was 0% among NT and 1.2% among NT- patients, whereas perioperative surgical complications were similar in both groups. One- and 5-year graft and patient survival were similar between the NT and PNT groups, but patients in the PNT group had significantly lower levels of hemoglobin and residual diuresis volumes at the time of transplant. Moreover, PNT patients had a longer pretransplant dialysis and a longer time on the waiting list.

**Conclusions:**

Simultaneous unilateral nephrectomy does not have a negative effect on patient and graft survival in patients with ADPKD and is associated with low morbidity. Pretransplant nephrectomy should be restricted only to highly symptomatic patients, whereas unilateral nephrectomy in asymptomatic patients should be performed during kidney transplantation only if massive kidney size precludes graft positioning.

## Introduction

Autosomal dominant polycystic kidney disease (ADPKD) is one the most frequent cause of end-stage renal disease (ESRD) in Europe and USA and may affect 2.5%-10% of dialysis patients. ADPKD is characterized by the development and progressive expansion of multiple bilateral cysts in the renal parenchyma, leading to a progressive decline in renal function owing to compression of normal functioning parenchyma by enlarging cysts, with renal failure or ESRD occurring in up to 70% of affected patients [1–4].

A massively enlarged polycystic kidney may cause discomfort to patients, and most recent data suggest that approximately one-fifth of ADPKD patients will require unilateral or bilateral nephrectomy [3,4]. However, many controversies exist concerning the need, indication, and timing for native nephrectomy in ADPKD patients scheduled for kidney transplantation [2,3,5–10]. The indications for pretransplant nephrectomy include ongoing hematuria, pain or discomfort, gastrointestinal symptoms such as early satiety, and risk of malignancy [3–5]. However, pretransplant nephrectomy is associated with increased morbidity and mortality in patients with ESRD, may deprive patients of residual diuresis, and may cause overall worsening of symptoms or renal failure [3,4,8,9,11]; hence, routine pretransplant nephrectomy is no longer recommended [4,5,11–15].

Some studies have argued that the sandwich technique, in which the more severely affected kidney is removed before transplantation and the remaining one after transplantation, may be the safest strategy [10,16]. Although this strategy may overcome the need for intraperitoneal access, usually associated with bilateral nephrectomies, this technique implies the need for 3 surgical interventions. Therefore, a nephrectomy simultaneous to kidney transplantation reduces the total number of procedures and is associated with a low risk of post-transplant complications, such as bleeding or infections, thereby increasing patient comfort [6,7,9,10,11–18].

The aim of the present study was to evaluate the feasibility of unilateral nephrectomy simultaneous to kidney transplantation and to determine the effect of this procedure on perioperative morbidity and mortality.

## Patients and Methods

From January 2000 to May 2015, at the Vascular Surgery and Organ Transplant Unit of the University Hospital of Catania, 680 kidney transplants were performed (572 from deceased donors and 108 from living donors).

Of these, ADPKD was the cause of ESRD in 145 patients (21.3%). The diagnosis of ADPKD was made on the basis of medical history, physical examination, ultrasonography, computed tomography scan, and in case of simultaneous nephrectomy, was confirmed by histologic examination. All polycystic patients underwent a complete physical examination and abdominal sonography to evaluate the size of polycystic kidneys. If native kidney volume did not allow for graft positioning, the patient was scheduled for simultaneous nephrectomy during kidney transplantation. If the kidney volume allowed the graft positioning, patients were evaluated on six-month basis by abdominal sonography. Pre-transplant nephrectomy was performed only in patients with recurrent bleeding requiring frequent blood transfusions, recurrent urinary tract infections, suspected kidney cancer, or significant gastro-intestinal symptoms. Final decision on the need for simultaneous nephrectomy was made at the time of kidney transplantation after re-evaluation with abdominal sonography.

This series included 127 kidney transplantations from deceased donors and 18 from living donors. The patients involved in this study gave a written informed consent authorizing all procedures. The Ethic Committee of University of Catania authorized the study and all procedures described in the manuscript; however, they represent the common clinical practice at our Institution, the study did not involve any experimental treatment, and does not reveal any confidential information about patient that could violate their privacy. The Italian Ministry of Health and Centro Nazionale Trapianti, which coordinates all transplant programs in Italy, moreover, authorized all the procedures. None of the transplant donors were from a vulnerable population and all donors or next of kin provided written informed consent that was freely given.

In 40 patients (27.5%) with ADPKD, a simultaneous native nephrectomy was performed together with kidney transplantation (group NT). Among these, 7 patients received dual kidney transplantation.

The surgical technique involved unilateral nephrectomy before kidney transplantation in all cases. Nephrectomy was performed on the right side, regardless of kidney size. Left nephrectomy was indicated in those patients who complained of greater discomfort on the left side or in re-transplantation where the right iliac fossa had already been used for the first transplantation. The procedure began with a Gibson incision with cranial extension up to the costal margin. After sliding the peritoneum, the polycystic kidney was identified and the duodenum was Kocherised to identify the renal hilum, which was isolated and dissected. The renal vessels and ureters were clamped, divided, and sutured with a polypropylene running suture. Renal transplantation was then performed using the standard technique for single- or ipsilateral dual-kidney transplantation [19]. No patient received a bilateral nephrectomy.

The indication for nephrectomy in most patients was the size of the native kidney with reduced space for kidney transplantation. Additional criteria were recurrent infections (in 2 patients) and recurrent bleeding from the native kidney (in 3 patients).

Patients of group NT were compared with 80 patients with ADPKD undergoing transplant without concomitant nephrectomy (group NT-). Moreover, clinical outcomes of patients of group NT were compared with 25 patients with ADPKD who underwent native nephrectomy before transplantation (group PNT). Clinical outcomes included time on dialysis, type of renal replacement therapy (hemodialysis or peritoneal dialysis), time on the waiting list, residual diuresis, hemoglobin level, albumin level, and survival of both the graft and the patient. Immunosuppression was based on a 3-drug regimen. Induction therapy with antithymocyte globulin (ATG-Fresenius, Fresenius, Bad Homburg, Germany) or anti-interleukin-2 receptor antibodies (Simulect, Novartis, Basel, Switzerland) was used in all groups for patients aged <55 years, those receiving a second transplant, or patients with panel-reactive antibodies >30% or with donor-specific antibodies (mean fluorescence intensity >3000). In the immunosuppression protocol, prednisolone was initiated at transplantation, with a starting dose of 500 mg of methylprednisolone and then tapered to a maintenance dose of 5 mg/d by the end of 4 months. Mycophenolic acid was given at a dose of 1440 mg/d. For patients receiving tacrolimus-based immunosuppression, tacrolimus was initiated at 0.1 mg/kg/d, with the dose adjusted to keep the levels at 10–12 ng/mL in the first month after transplantation and 8–10 ng/mL subsequently. For recipients receiving cyclosporine-based immunosuppression, cyclosporine was started the day after transplantation at 5 mg/kg/d, with the dose adjusted to keep the level at 180–200 ng/mL for the first 3 months after transplantation, and 120–180 ng/mL for 3–6 months after transplantation. Everolimus was initiated at 1.50 mg/d beginning within 5 days after transplantation, with the dose adjusted to keep the level at 3–6 ng/mL. Everolimus was not preferentially given to patients with ADPKD.

Rejection therapy consisted of steroid pulses of 500 mg of methylprednisolone for 3 days. Delayed graft function was defined as the need for dialysis in the first week after transplantation. Doppler ultrasonography was performed daily, starting immediately after transplantation.

### Statistical analysis

Values are expressed as means ± standard error of the mean, unless otherwise stated. Parametric data were analyzed using the t-test, whereas quantitative data were analyzed using the chi-square test. Patients and graft survival were calculated using the Kaplan-Meier method. Comparisons of patient characteristics and perioperative complication rates between groups were made using the continuity-adjusted chi-square test. A value of p < .05 was considered statistically significant. Perioperative complications and patient death were defined as events occurring within 30 days after transplantation. The propensity score for each patient was calculated by a logistic regression model, using the following characteristics as covariates: donor age, recipient age, pre-transplant dialysis, cold ischemia time, and duration of delayed graft function. A one-to-one match between the two groups was obtained by use of the nearest neighbor matching with the caliper width of 0.01. All statistical analyses, including propensity-score matching, were performed using SPSS (IBM SPSS Statistics for Windows, Version 22.0).

## Results

Clinical characteristics of the study population are reported in Table 1. First, we compared the clinical outcomes of the NT group with the NT- group.

**Table 1:** Clinical data of 40 kidney transplant recipients with concomitant unilateral nephrectomy (NT). Patients of group NT were compared with 80 polycystic patients not undergoing simultaneous nephrectomy during kidney transplantation (NT-) and with 25 patients undergoing nephrectomy before transplantation (PNT).

Characteristics	NT	NT(-)	*P* ValueNT vs NT-	PNT	*P* valueNT vs PNT
**N**	40	80		25	
**Age (yrs)**	51 ± 9.7	52 ± 10	.638	44 ± 8	.422
**Sex (M/F)**	26/14	50/30	.732	15/10	.648
**BMI (Kg/m**^**2**^**)**	26.5 ± 5	27.3 ± 4.2	.443	25.6 ± 3.1	.335
**Waiting list (months)**	17.1 ± 21.1	19.4 ± 22.1	.824	24.5 ± 18.3	**.04**
**Pre-transplant dialysis (months)**	27.4 ± 34.1	39.6 ± 34	.108	42.1 ± 28	**< .001**
**Hemodialysis/peritoneal dialysis**	23/11	70/7	.224	25	**< .001**
**Pre-emptive transplantation (n)**	7	3	.921	0	**.04**
**Second transplant (n)**	1	5	.886	0	
**Living Donor (n)**	5	8	.975	5	.884
**Deceased Donor (n)**	35	72		20	
**DKT (n)**	7	7		0	
**Donor age (yrs)**	56.7 ± 16	53.1 ± 15.7	.264	55.2 ± 12.4	.235
**Residual diuresis (ml)**	1254 ± 327	622 ± 118	**.004**	280 ± 92	**< .001**
**Pre-transplant Hb (g/dL)**	11.4 ± 3.2	9.2 ± 2.2	**.022**	9.0 ± 1.82	**< .001**
**Albumin level (g/dL)**	4.1 ± 1.5	3.8 ± 1.6	.664	2.9 ± 1.2	**.04**
**Indications to nephrectomy (n,%)**					
Creating space for graft positioning	39 (97.5)			21 (84)	
Recurrent urinary tract infections	0 (0)			1 (4)	
Recurrent hematuria	1 (2.5)			2 (12)	
**Cold ischemia time (min)**	900 ± 370.2	940 ± 376.1	.616	895 ± 395.1	.883
**Operative Time (min)**	193.2 ± 71.7	157.9 ± 46.1	**.03**	165 ± 44.5	**.05**
**Immunosuppression (n)**					
Induction	18 (45%)	38(42.2%)	.224	9 (36%)	.445
Tac+MPA+ Ster	30 (75%)	65 (81.2%)	.456	19 (76%)	.118
CyA+MPA+Ster	3 (7.5%)	5 (6.2%)	.765	2 (8%)	.823
Ever+Tac+Ster	7 (17.5%)	10 (12.5%)	.833	4(16%)	.553

BMI: body mass index;DKT:dual kidney transplantation; Hb: Hemoglobin; TAC:tacrolimus; MPA: mycophenolic acid; Ster: steroids; CyA: cyclosporine; EVE:everolimus.

The mean follow-up of patients was 5.6 years (range, 0.8–14.7 years). Simultaneous nephrectomy was performed in the right side in 31 patients and in the left side in 9 patients.

The mean weight of the removed kidneys was 1908 g (range, 800–5300 g), whereas the mean cranial-caudal diameter was 24.1 cm (range, 16.5–32 cm), as confirmed by the pathologist’s report. Recipient and donor characteristics were similar between the 2 groups (Table 1). Patients in the NT group were more frequently receiving peritoneal dialysis, had a lower time on dialysis and on the waiting list, and underwent a pre-emptive transplantation more frequently compared with NT- patients, without reaching the level of statistical significance. Of interest, cold ischemia time, acute rejection episodes, hospital stay, and blood loss did not increase in patients undergoing simultaneous nephrectomy: Operative time was increased by 20–40 min depending on the size of the native kidney in the NT group (*P =* .03). No patient in the NT group died in the perioperative period, whereas 1 patient in the NT- group died due to pneumonia 24 days after the operation (Table 2).

**Table 2:** Surgical complications and clinical outcomes of 40 kidney transplant recipients with concomitant unilateral nephrectomy (NT). Patients of group NT were compared with 80 polycystic patients not undergoing simultaneous nephrectomy during kidney transplantation (NT-) and with 25 patients undergoing nephrectomy before transplantation (PNT).

Characteristics	NT	NT(-)	*P* ValueNT vs NT-	PNT	*P* valueNT vsPNT
**N**	40	80		25	
**Surgical complications (n)**	3 (7.5%)	10 (12.5%)	.098	5 (21%)	.224
wound infection	0	2	.543	2	
incisional hernia	1	3	.218	1	
Lymphocele	1	3	.388	2	
Renal Vein Thrombosis	1	0	.457	0	
**Blood Loss (ml)**	300 ± 122	522 ± 142	.221	421 ± 138	.314
**DGF**	9 (22.5%)	26 (32.5%)	.167	11 (44%)	**.03**
**Duration of DGF (days)**	11.7 ± 3.4	14.3 ± 7.7	.338	16.3 ± 5.2	**.022**
**Acute rejection**	1(2.5%)	2 (2.5%)	.432	0	.324
**Hospital stay (days)**	13.3 ± 6.1	16.5 ± 16.5	.321	15.4 ± 12.5	.662
**Mean 1-year Serum Creatinine (mg/dl)**	1.58	1.55	.842	1.6	.444
**Mean 5-year Serum Creatinine (mg/dl)**	1.60	1.65	.795	1.6	.554
**Graft Survival 1y**	95%	93%	.903	96%	.665
**Graft Survival 5y**	87.5%	76.2%	.544	84%	.641
**Patient Survival 1y**	100%	97%	.288	100%	.324
**Patient Survival 5y**	100%	92%	.128	92%	.335

DGF: delayed graft function.

**Fig 1:**
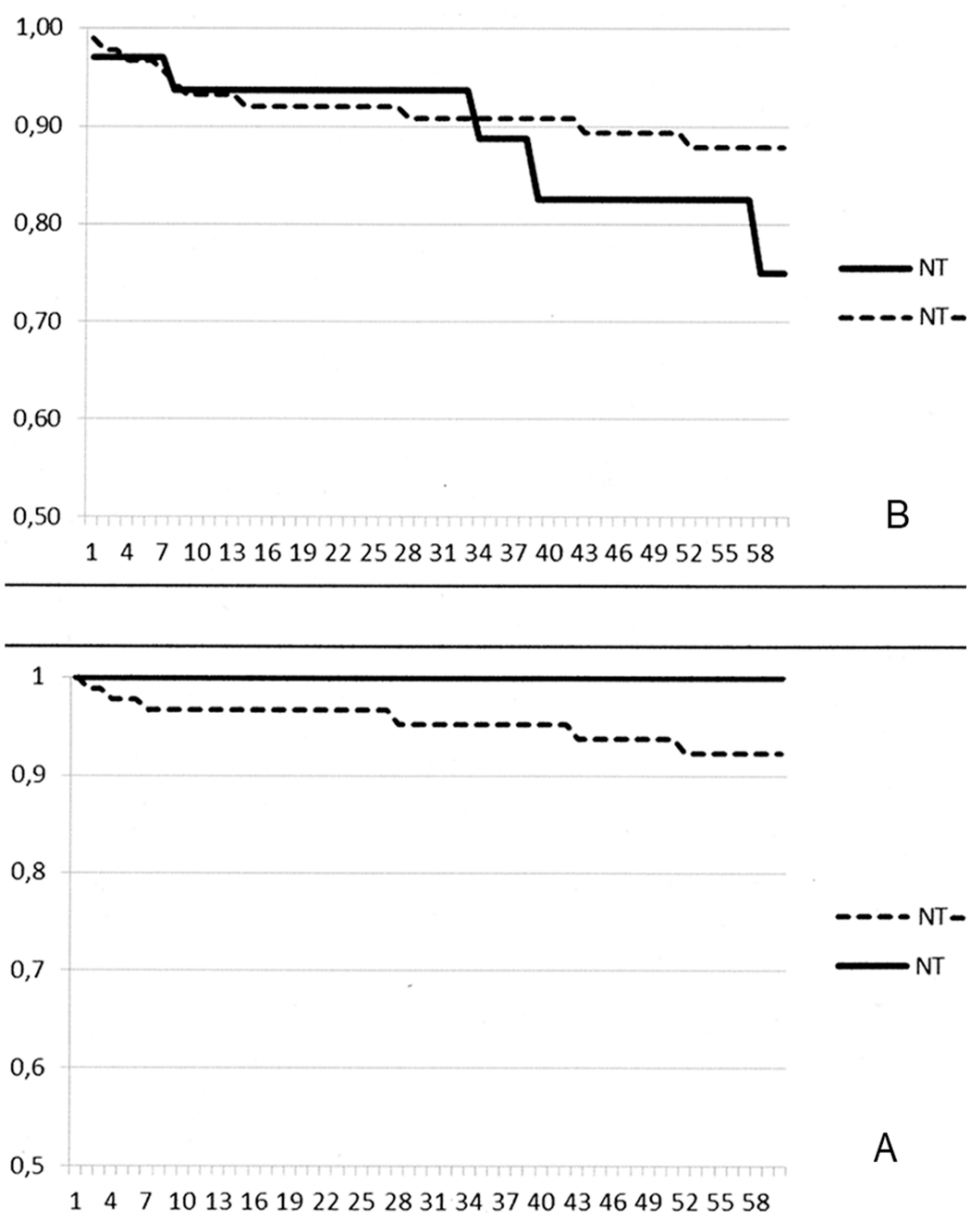
Post-transplant outcomes of 40 kidney transplant recipients with autosomal dominant polycystic kidney disease with simultaneous ipsilateral nephrectomy (group NT) compared with 80 recipients with polycystic disease who did not receive adjuvant nephrectomy (group NT-). (A) Patient survival and (B) graft survival.

There were no significant differences in post-transplant outcomes between the 2 groups. One- and 5-year patient survivals were both 100% in the NT group and 97% and 92% in the NT- group, respectively (Fig 1); one- and five-year graft survivals were 95%, and 87.5% in the NT group and 93% and 76.2% in NT- group (Fig 1). During follow-up, 3 patients in the NT group and 6 patients in the NT- group underwent a kidney re-transplantation.

There were no vascular complications in the NT- group, whereas 1 patient in the NT group experienced a renal vein thrombosis on postoperative day 7, requiring the graft nephrectomy. Two patients in the NT- group required a reoperation for a post-transplant hematoma. There was no significant difference in the incidence of wound infection, lymphocele, or incisional hernia between the 2 groups.

One kidney neoplasm was observed in the NT group: In a 55-year-old man, the histological examination of the removed kidney revealed an incidental 2-cm, grade 2 Fuhrman papillary renal carcinoma (pT1a). This patient underwent prophylactic contralateral nephrectomy 1 month after kidney transplantation, and no renal neoplasm was detected in the removed kidney. At 7-year follow-up, the patient is in good health, with a well-functioning kidney and no sign of cancer recurrence.

There were no significant differences in 1-year (1.58 vs. 1.55 mg/dL; *P* = .842) and 5-year (1.60 vs. 1.65 mg/dL; *P =* .795) mean serum creatinine levels between the 2 groups.

After propensity matching, there were 34 patients in the NT group and 34 in the NT- group with correction of all differences in baseline characteristics (Table 3). Again, there were no significant differences in post-transplant outcomes between the 2 groups. One- and 5-year patient survivals were both 100% in the NT group and 100% and 94.1% in the NT- group, respectively; one- and five-year graft survivals were 97.1%, and 88.2% in the NT group and 94.1% and 91.2% in NT- group (Table 4).

**Table 3:** Demographic and baseline characteristics after propensity score matching.

Characteristics	NT	NT(-)	*P* Value
**N**	34	34	
**Age (yrs)**	51.74	52.50	0.724*
**Donor Age (yrs)**	54.15	56.56	0.485*
**Pre-transplant Dialysis (months)**	22.29	23.97	0.745*
**Cold Ischemia time (min)**	882.35	922.94	0.634*
**Duration of DGF (days)**	4.50	4.18	0.864*

Values are expressed as means. DGF: delayed graft function.

**Table 4:** Clinical outcomes after propensity score matching.

Characteristics	NT	NT(-)	*P* Value
**N**	34	34	
**Graft survival 1 y**	97.1%	94.1%	1
**Graft survival 5 y**	88.2%	91.2%	1
**Patient Survival 1 y**	100%	100%	1
**Patient Survival 5 y**	100%	94.1%	1

Twenty-five kidney transplant recipients had undergone pretransplant nephrectomy (PNT): 20 patients underwent bilateral nephrectomy and 5 patients unilateral nephrectomy.

The indications for pretransplant nephrectomy were recurrent hematuria needing multiple blood transfusion (3 patients), recurrent urinary tract infections (1 patient), and creating space for the kidney graft in asymptomatic patients (21 patients).

Compared with NT patients, PNT patients were more likely to receive hemodialysis (*P<* .001) and had longer pretransplant dialysis (*P<* .001) and waiting list time for kidney transplantation (*P =* .04). Moreover, PNT patients had a significantly lower residual diuresis volume (*P <* .001) and hemoglobin level at transplant (*P <* .001, Table 1).

There were no significant differences in graft and patient survival rates between the 2 groups: 1-year patient survival was 100% for both the NT and PNT groups, whereas 5-year patient survival was 100% and 92% in NT and PNT groups, respectively. One-year graft survival was 95% in the NT group and 100% in the PNT group (*P =* .665), whereas 5-year graft survival was 87.5% in the NT and 84% in the PNT group (*P =* .664). Graft function was similar between the 2 groups: the 1-year mean serum creatinine level was 1.58 mg/dL and 1.6 mg/dL in the NT and PNT groups, respectively, whereas the 5-year mean serum creatinine level was 1.60 mg/dL in the NT group and 1.62 mg/dL in the PNT group. Taken together, these data strongly suggest that patients who had undergone simultaneous nephrectomy had similar graft and patient outcomes as those who had undergone a pretransplant nephrectomy. However, patients in the PNT group had more discomfort due to a severe anephric/anuric state, as suggested by the lower levels of hemoglobin before transplantation and the lower residual urine volume.

One of the main concerns regarding the use of ipsilateral simultaneous nephrectomy is the likelihood of the need for contralateral nephrectomy because of infections, cyst enlargement, and cancer. However, contralateral nephrectomy after transplantation was performed in only 8 of all ADPKD patients (6.6%): 3 to allow for positioning of a second transplantation, 3 due to dyspnea and early satiety, 1 for hematuria, and 1 for recurrent urinary infections.

## Discussion

This single-center matched study includes a large series of patients who underwent kidney transplantation with simultaneous single nephrectomy. Patients who had undergone simultaneous nephrectomy were analyzed retrospectively for the most significant parameters that could affect outcome after transplantation with a matched cohort of polycystic kidney transplant recipients who did not undergo a simultaneous native nephrectomy.

Kidney transplantation is undoubtedly the best replacement therapy for ADPKD patients with ESRD, but there is still an ongoing discussion about the indications for, timing of, and approach to native nephrectomy. Although some older studies reported a significantly better graft function and postoperative survival for patients undergoing pretransplant unilateral or bilateral nephrectomy [20,21], many recent publications suggest that pretransplant nephrectomy may be associated with a higher rate of morbidity and mortality [4,7,11,22,23]. Therefore, routine pretransplant nephrectomy is no longer recommended [4,5,8–12,14,15] and should be performed only when clearly indicated. In our clinical practice, pretransplant nephrectomy is performed only in patients with recurrent bleeding, infection, early satiety, or suspected malignancy, whereas native nephrectomy simultaneous to kidney transplantation is indicated if massive kidney size does not allow for graft positioning.

Although some studies [20,21] suggested that pretransplant nephrectomy has the advantages of reduced risk of bleeding events and infectious complications, as well as relief of patient’s subjective complaints of lumbar pain, more recent studies suggested that simultaneous nephrectomy does not increase the rate of post-transplant complications and does not affect patient and graft outcomes [5,7,10,22,24]. In the present series, we found no differences in patient survival or kidney function rates in patients who underwent simultaneous nephrectomy compared with matched patients undergoing transplant for ADPKD without nephrectomy, even after propensity score matching. Simultaneous nephrectomy resulted in a very low rate of post-transplant complications but not significantly different post-transplant morbidity compared to that of matched patients with ADPKD undergoing transplant without nephrectomy.

Operative time was higher in the NT group (193.2 ± 71.7 vs. 157.9 ± 46.1 minutes; *P* = .03), with an increase of 20–40 minutes, depending on the size of the kidney. Similar findings were reported by Lucas et al. [17], who reported a difference of approximately 30 minutes, and Neef et al. [6], who reported an operative time 20–45 minutes longer in patients who underwent simultaneous nephrectomy. The operative time was, however, significantly shorter of that needed for simultaneous bilateral nephrectomy and kidney transplantation, where a total operative time as long as 7.5 h was reported [7,12,14,18].

Of interest, a slightly longer operative time did not correspond to a longer cold ischemia time: In our series, there was no significant difference between the 2 groups, with a shorter time in the NT group (900 ± 370.2 vs. 940 ± 376.1 minutes; *P* = .616).

The potential disadvantages of pretransplant nephrectomy are rendering patients into an anephric and anuric state, potentially resulting in a lower survival [25]. Patients with a polycystic kidney left in situ have the advantages of maintaining urine output that makes the fluid restrictions on dialysis easier to handle and retaining the kidney’s ability to produce erythropoietin, thus maintaining higher hemoglobin values than other patients with chronic renal disease [9].

This study confirmed this hypothesis: patients undergoing simultaneous nephrectomy and kidney transplantation had significantly higher hemoglobin levels and residual diuresis at the time of transplant compared with patients who had undergone nephrectomy before transplantation. Moreover, patients in the NT group had less time spent on the waiting list, reduced time on dialysis, more frequent use of peritoneal dialysis as replacement therapy, and more frequently underwent a pre-emptive transplantation. Taken together, these results suggest that a concurrent nephrectomy at the time of transplantation may avoid or reduce complications related to the anephric/anuric state, improving the clinical status of patients scheduled for transplantation and post-transplant outcomes, as a result of a shorter time on dialysis and on the waiting list [26].

In patients with significant co-morbidities, peri-operative risk may be increased by adding the simultaneous nephrectomy of a large kidney, so that in such situations the decision to perform the native nephrectomy should be adapted on a case-by-case basis.

No patient died in the perioperative period in the NT group, and patient and graft survival rates and graft function did not differ between the groups, confirming that simultaneous nephrectomy does not increase the rate of post-transplant complications and does not affect patient or graft outcomes [5–7,10,17,20,22,24,25,27,28].

Of interest, graft and patient survival and graft function of NT patients were not different from those of patients who had undergone pretransplant nephrectomy (group PNT), suggesting, again, that pretransplant nephrectomy is not associated with better post-transplant outcomes, but patients had more severe complications related to the anephric/anuric state. Indeed, PNT patients had significantly lower hemoglobin levels and residual urine volume at transplantation, were more likely to receive long-term hemodialysis rather than peritoneal dialysis as replacement therapy, and had a longer time on the waiting list for kidney transplantation.

Bilateral nephrectomy with concurrent kidney transplantation has been suggested by some authors to reduce the incidence of post-transplant complications related to the retained kidney, such as bleeding, recurrent infections, and cancer. Although this procedure may be well tolerated with a high patient contentment, bilateral nephrectomy is associated with an increased risk of post-transplant complications [10,14,17,24,29,30–34].

Otherwise, a unilateral nephrectomy with concurrent transplantation might be sufficient to alleviate symptoms and to provide room for kidney transplantation. Moreover, the volume of native kidneys in patients with ADPKD significantly decreases after successful transplantation. Yamamoto et al. [35] showed that native kidney volume was reduced by a mean of 38% at 1 year in all but 1 of the 33 patients with ADPKD after transplantation, and much of this decrease was observed during the first year after transplantation. We were not able to evaluate the change in volume of native kidneys after transplantation, but only 8 ADPKD patients (6.6%) required a native nephrectomy after transplantation, most often to allow the space for a second transplantation.

Another drawback of leaving the polycystic kidneys in situ is the risk of cancer. However, the overall risk of finding an incidental renal neoplasm is not different in ADPKD patients compared with the general population [36], and these tumors can be completely treated by partial or radical nephrectomy [37].

In this study, one patient had an incidental low-grade papillary renal cancer in the removed polycystic kidney. However, no cancer was detected in the contralateral kidney after removal, and no signs of cancer recurrence were demonstrated up to 7 years after transplantation. These data, overall, suggest that the need for post-transplant native nephrectomy of the remaining kidney(s) in ADPKD patients is very low.

To our knowledge, this is the first that compares outcomes of simultaneous nephrectomy and kidney transplantation versus transplantation alone and versus pre-transplant nephrectomy and delayed transplantation in a population of patients with ADPKD. The results strongly suggest that simultaneous nephrectomy does not adversely affect clinical outcomes in patients with ADPKD after kidney transplantation and does not increase the rate of surgical complications.

Although the study reported such important findings, we are conscious of its limitations. It is retrospective in nature but is based on a large homogeneous population of patients who underwent kidney transplantation in a single center. The study sample is relatively small, but a better-powered study would require a study population of at least 400 patients in each group, which is not likely to be performed [7]. Moreover, patients undergoing a simultaneous nephrectomy may require a life-long imaging follow-up of the remaining kidney.

## Conclusions

Simultaneous unilateral nephrectomy does not have a negative effect on patient and graft survival in patients with ADPKD. The findings of this study support a restrictive use of pre-transplant native nephrectomy in asymptomatic patients. Simultaneous nephrectomy does not increase post-transplant surgical complications; the patient may benefit from the residual diuresis and kidney function while waiting for a kidney transplantation, the risk of cancer in the remaining kidney is extremely low, and the volume of remaining kidney is expected to decrease. Taken together, these results suggest that simultaneous nephrectomy and kidney transplantation should be preferred to pretransplant nephrectomy in asymptomatic patients with ADPKD listed for transplantation, and nephrectomy should be requested only if massive kidney size prevents graft placement.
